# Fermentation Kinetics and Changes in Levels of Antinutrients in Pearl Millet and Pearl Millet‐Maize Composite Dough Recipes Used to Prepare *Injera*

**DOI:** 10.1002/fsn3.70598

**Published:** 2025-07-09

**Authors:** Tadesse Fenta Yehuala, Minaleshewa Atlabachew, Mohamad Farshard Aslam, Lara Allen, Howard Griffith, Jane L. Ward, Peter R. Shewry, Anastasia Kanellou, Wanjiku Gichohi‐Wainaina, Helen Walle Endalew, Metadel Kassahun Abera, Mesfin Wogayehu Tenagashaw, Gizaw Desta Gessesse, Hirut Assaye Cherie

**Affiliations:** ^1^ Faculty of Chemical and Food Engineering Bahir Dar Institute of Technology, Bahir Dar University Bahir Dar Ethiopia; ^2^ Department of Analytical Chemistry College of Science, Bahir Dar University Bahir Dar Ethiopia; ^3^ Department of Nutritional Sciences School of Life Course and Population Sciences, King's College London London UK; ^4^ Centre for Global Equality Cambridge UK; ^5^ Department of Chemical Engineering and Biotechnology University of Cambridge Cambridge UK; ^6^ Department of Plant Sciences University of Cambridge Cambridge UK; ^7^ Rothamsted Research Harpenden UK; ^8^ Department of Food Science and Technology University of West Attica Athens Greece; ^9^ WorldFish Penang Malaysia; ^10^ International Crops Research Institute for Semi‐Arid Tropics (ICRISAT) Lilongwe Malawi; ^11^ International Crops Research Institute for Semi‐Arid Tropics (ICRISAT) Addis Ababa Ethiopia

**Keywords:** microbial quality and fermentation, pearl millet, phytate, raffinose

## Abstract

While pearl millet is rich in important nutrients with potential health and nutrition benefits, it contains antinutrients that limit the bioavailability of minerals and the digestibility of starches and proteins; however, fermentation is believed to reduce these antinutrient levels. The objective of this work was to determine the fermentation kinetics and its implications for changes in the levels of antinutrients in pearl millet and pearl millet‐maize composite dough recipes used to prepare *Injera*, a traditional fermented flatbread consumed in Ethiopia. Three dough recipes identified through focus group discussion with women from the Dangeshita sub‐district, Dangila District, Ethiopia, were investigated: pure pearl millet dough (P), a 1:1 mixture of pearl millet and maize (P1M1) and a 1:2 mixture of pearl millet and maize (P1M2) doughs. Significant decreases in pH were observed for all dough recipes at the later stages of fermentation. This drop in pH was accompanied by a rapid increase in titratable acidity. Counts of aerobic mesophilic bacteria and molds decreased (with molds reaching zero), while counts of yeasts and lactic acid bacteria (LAB) increased at the later stage of fermentation across all dough recipes. A two‐step fermentation process characterized by both lactic acid and alcoholic fermentation was identified, yielding lactic acid and mannitol as primary end products. Phytate was degraded by 91.3% in pearl millet (P) dough, by 98.2% in P1M1 dough, and by 72.7% in P1M2 dough after 168 h (7 days) fermentation. All fermented dough recipes resulted in reduced levels of raffinose at the later stages of fermentation, with the highest degradation noted in pearl millet (P) dough (95%) followed by P1M1 dough (87.7%) and P1M2 (80.8%) dough. In conclusion, 7 days fermentation resulted in significant reductions of phytate and raffinose levels in all dough recipes.

## Graphical abstract

Fermentation time influenced the pH, metabolite profiles, and microbial counts of pearl millet‐based injera sourdough. This process facilitates the degradation of antinutrients, specifically phytate and α‐galactosides (raffinose). Notably, phytate degradation was higher in pearl millet sourdough compared to other recipes, which has direct implications for the bioavailability of minerals, protein, and starch. Additionally, fermentation changes the monosaccharide, disaccharide, and organic acid profiles during fermentation.

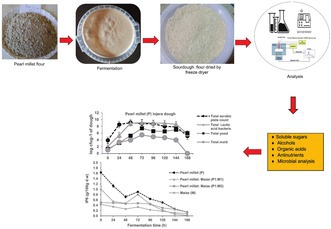

## 1 Introduction

*Injera* is a traditional fermented flatbread consumed in Ethiopia, accounting for approximately 70% of the caloric intake (Ashagrie 2012). It is prepared from various cereal flours such as teff, sorghum, wheat, rice, maize, millet, and barley (Mihrete and Bultosa 2017; Neela and Fanta 2020). Cereals are known to contain antinutrients which reduce the bioavailability of minerals and the digestibility of starch and proteins. On the other hand, mineral deficiencies, especially of iron, zinc, and calcium, are highly prevalent among the Ethiopian population, largely due to the daily consumption of cereal‐based foods and limited consumption of animal‐based foods. Studies indicate that a significant portion of the Ethiopian population, notably women and children, experiences inadequate intake of these essential nutrients, which can lead to serious health consequences, including anemia and impaired growth (Belay et al. 2022; Abdu et al. 2022).

Fermentation of cereals has been shown to reduce antinutrients due to the activation of enzymes which break down these compounds (Adebo et al. 2022; Osman 2011). On the other hand, the activation of these enzymes is dependent on fermentation kinetics, which are influenced by the specific raw materials utilized (Adeyemo and Onilude 2013). Few studies have reported on how the raw materials used in *Injera* preparation influence fermentation kinetics and the reduction of antinutrients. For example, Baye et al. (2013) investigated the effects of teff–white sorghum (TwS), barley–wheat (BW), and wheat–red sorghum (WrS) flour blends on fermentation kinetics, finding complete hydrolysis of phytate in WrS and BW *Injera*, while TwS *Injera* showed a 28% hydrolysis of phytate.

An improved variety of pearl millet called Kola‐1 has been introduced for cultivation in the arid regions of Ethiopia as a food and nutrition security crop (Berhanu et al. 2020) associated with its resilience to harsh environmental conditions (Satyavathi et al. 2021) and the crop is particularly valued for its suitability for the preparation of *Injera*. With its relatively high protein content (ranging from 9% to 24%) (Ali et al. 2003) and being rich in minerals such as iron, zinc, calcium, copper, potassium, manganese, and phosphorous (Yadav et al. 2014; Gowda et al. 2022), pearl millet possesses a high potential to provide nutrition security. On the other hand, pearl millet, like other cereals, contains antinutrients such as phytic acid, tannin, phenols, and α‐galactosides that notably diminish the bioavailability of nutrients (Rani et al. 2018). Therefore, this study aimed to investigate the preparation of *Injera* from pearl millet dough and pearl millet‐maize composite dough recipes based on practical observations and to determine the fermentation kinetics and its implications in changes in the levels of antinutrients.

## 2 Materials and Methods

### 2.1 Raw Materials

Pearl millet (Kola‐1 variety, accession number: ICMV221) was obtained from Sekota Dryland Agricultural Research Center, Ethiopia. Maize (BH 660) was obtained from targeted farmers in Dangila district (Ethiopia) in consultation with development agency experts operating in the area who are providing agricultural extension services directly to the farmers.

### 2.2 Processing of Raw Materials

Both grains (5 kg of pearl millet and 5 kg of maize) were cleaned individually to remove any extraneous materials such as stones, dust particles, husk, undersize, and immature grains. The cleaned grains were then sun dried and separately milled using a laboratory hammer mill (TPS‐JXFM110, China), and sieved to 500 μm. Flour samples were stored at 4°C for further analysis.

### 2.3 Preparation of Dough

**FIGURE 1:**
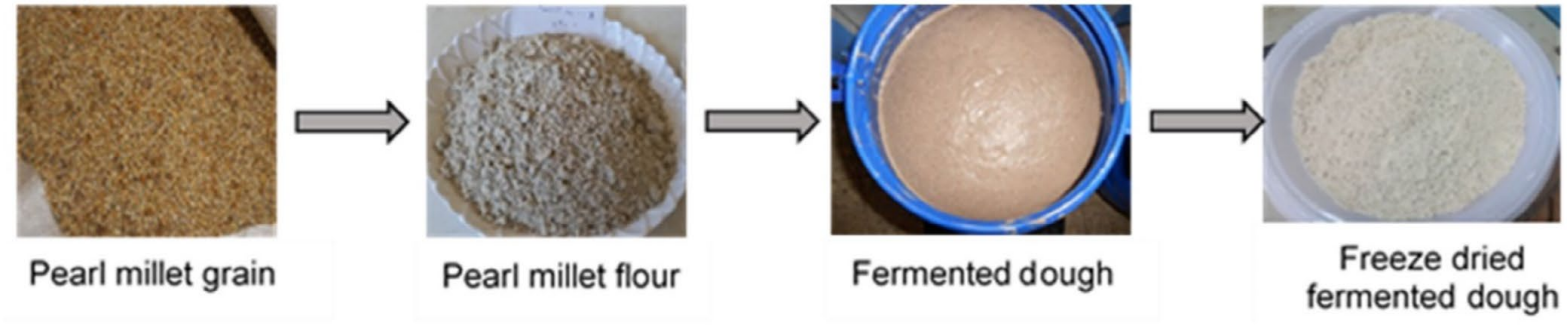
Fermented dough preparation from pearl millet flour.

Pearl millet‐based *Injera* dough preparation and fermentation were conducted at the Food Processing Laboratory, Faculty of Chemical and Food Engineering, Bahir Dar University, Ethiopia (Figure 1). The recipes and dough preparation methods were based on the traditional millet *Injera* dough preparation and fermentation methods practiced by women from the Dangeshita sub‐district, Dangila District, Ethiopia, a community known for millet and maize production and consumption. Focus group discussions were conducted with women from this community, and three *Injera* preparation recipes were identified: *Injera* from pearl millet alone, mixing pearl millet and maize in a one to one ratio (P1M1) and mixing pearl millet and maize in a one to two ratio (P1M2). For the P1M1 (1 pearl millet: 1 maize) recipe, maize dough was initially prepared and fermented for 3 days. Then, an equal amount of pearl millet was added to the mixture on the third day and left to ferment until the baking day. For the P1M2 (1 pearl millet: 2 maize) recipe, cleaned pearl millet and maize grains were mixed in a 1:2 ratio and milled together. The composite flour was then mixed with water (1flour: 1 water) to prepare the dough and left to ferment for 3 days. On the third day, an equal amount of composite flour (P1M2) (1 flour: 3 water) was added and left to ferment until the baking day. Baking the dough into *Injera* is usually done after the fourth fermentation day and can be left until the seventh day, depending on the needs of the family for *Injera*. In this study, the doughs from the different recipes were fermented naturally for 168 h (7 days) at room temperature (around 22°C), and dough samples were collected at 24 h intervals and freeze‐dried using a Scanvac Cool Safe 55–9 (Scanvac, Denmark). The dried samples were finely ground and stored at −20°C in sealed polyethylene bags for subsequent laboratory analyses. Samples for metabolite analysis were transported under refrigerated conditions to the Rothamsted Research, Harpenden, Hertfordshire AL5 2JQ, UK.

### 2.4 Microbiological Analysis

The microbiological profile of pearl millet‐based dough during fermentation was assessed through the enumeration of total aerobic plate count (APC), lactic acid bacteria (LAB), total yeast, and mold at 24 h intervals, following standard methods (da Silva et al. 2018).

Total aerobic plate counts (APC) were determined as follows: Aseptic samples of 10 g of dough were homogenized in 90 mL of 0.1% peptone water. Under laminar flow conditions, serial dilutions were prepared, and 1 mL aliquots were spread onto pre‐solidified plate count agar (PCA; HiMedia M091) plates. After incubation at 35°C for 48 h, colonies were counted.

For the enumeration of LAB, the spread plate technique was employed. A 0.1 mL aliquot of the diluted sample was spread onto pre‐solidified de Man, Rogosa, and Sharpe (MRS) agar (Oxoid CM0361) plates and incubated anaerobically at 30°C for 48 to 72 h. Colony‐forming units (CFU) were then enumerated.

Yeast and mold counts were performed using standard procedures. Serial dilutions of the samples were spread onto potato dextrose agar (PDA; HiMedia M096) plates using a sterilized glass rod. To suppress bacterial growth, tartaric acid was added to the media. The plates were then incubated at 25°C for 5 days, after which yeast and mold colonies were counted as CFU per gram of sample and expressed as log CFU/g.

### 2.5 Fermentation Kinetics

#### 2.5.1 pH and Total Titratable Acidity

The pH of the various dough samples was determined by immersing a calibrated pH meter (Hanna Instruments pH 210, pH Meter, Romania) in homogenate dough (AOAC 1995). Total Titratable Acidity (TTA) of *Injera* dough samples was determined following the method described by Sadler (2010). Samples (10 g) were diluted in 100 mL of deionized water and titrated with 0.1 N NaOH to the endpoint at 24 h intervals. TTA was then expressed as percent lactic acid equivalent.

#### 2.5.2 Determination of Metabolites by ^1^H‐NMR Spectroscopy

Thirty milligrams of fermented dough flour samples were extracted at 50°C using 80:20 D_2_O:CD_3_OD containing 0.05% d4–trimethylsilyl propionate (TSP; 1 mL) as an internal standard (Shewry et al. 2022). The supernatant was removed after the extracted sample was centrifuged at 13,000 rpm for 5 min, and then the supernatant was heated to 90°C for 2 min to halt enzymatic activities. Cooling and centrifugation again, and the supernatant (650 μL) were transferred to an NMR tube (5 mm in size) for analysis. ^1^H‐NMR spectra were acquired under automation at 300°K using an Avance Neo Spectrometer (Bruker Biospin, Coventry, UK) operating at 600.0528 MHz, with a cryoplatform and 5 mm triple inverse probe. Spectra were collected using 16 scans of 65,536 data points with a spectral width of 7143 Hz. Water suppression pulse sequence with a 90° pulse and a relaxation delay of 5 s.

Fourier transformation of the spectra was performed using an exponential window function with a line broadening value of 0.5 Hz. Automated phasing and baseline correction procedures were then implemented. Automated reduction and processing of ^1^H‐NMR spectra were performed using Analysis of Amix (MIXtures software Bruker Biospin) to American Standard Code for Information Interchange (ASCII) files with integrated regions of 0.01 ppm width. Spectral intensities were scaled to the d4–TSP region (δ0.05 to −0.05). Signal intensities for major metabolites were extracted by comparing spectra to known standards run under similar conditions. Quantitation relative to the d4–TSP standard was achieved by normalizing the signal intensities of the characteristic peaks of each metabolite based on the number of hydrogens contributing to each peak.

#### 2.5.3 Phytate Analysis

The phytate content of unfermented and fermented dough samples was determined using a Megazyme (Megazyme‐KPHYT, Bray, Ireland) kit as per the protocol from the manufacturer (McKie and McCleary 2016). Fermented dough samples were subjected to hydrochloric acid digestion. Phytate (IP6), in the form of myoinositol phosphate, was extracted using phytase and alkaline phosphatase solutions. Following a modified colorimetric method, phosphorus release was quantified by measuring absorbance at 655 nm relative to a blank. Phytate content was then expressed as grams of IP6 per 100 g of sample.

### 2.6 Statistical Analysis

Triplicate determinations were made for all samples and parameters. Data were reported in mean ± standard deviation to estimate variation between treatments. Data were analyzed using the general linear model procedure in SPSS version 26. Analysis of variance (ANOVA) was used to assess statistical differences between treatment means. Significant variations (*p* < 0.05) were further tested using Tukey's post hoc test.

## 3 Results and Discussion

### 3.1 Microbial Counts During Dough Fermentations

#### 3.1.1 Total Aerobic Plate Count

**FIGURE 2:**
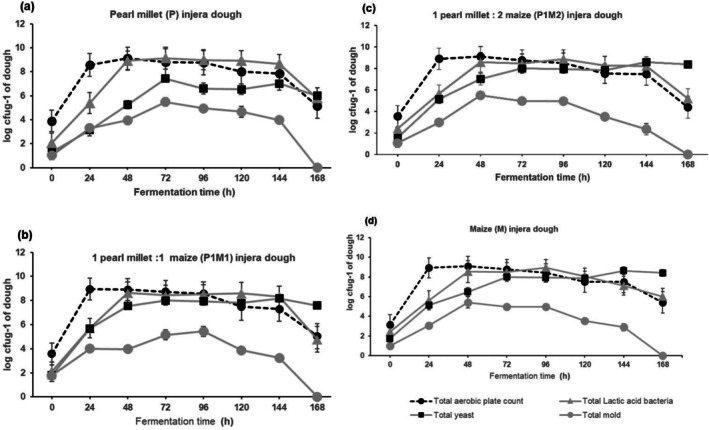
Changes in microbial counts during pearl millet‐based dough fermentations. Pearl millet dough (a), 1 pearl millet: 1 maize, P1M1 dough (b); 1 pearl millet: 2 maize, P1M2 dough (c) and maize dough (d). Error bars represent mean ± SD.

Changes in total aerobic plate counts among the different sourdough recipes are presented in Figure 2a–d. The total aerobic plate counts in all dough samples increased up to 72 h of fermentation, with values ranging from 3.16 to 9.11 log cfu/g. The increase in aerobic plate counts at the beginning of fermentation could be attributed to the availability of nutrients and favorable temperature, which enhance their metabolic activities and support their growth rates (Niu et al. 2025; Petrović et al. 2025). Notably, the total aerobic plate count of the fermented dough recipes decreased when the fermentation time was extended from 72 to 168 h. This decrease may be attributed to the accumulation of metabolites and the acidic conditions produced during the fermentation by lactic acid bacteria (Ravyts and De Vuyst 2011).

#### 3.1.2 Counts of Lactic Acid Bacteria

The counts of lactic acid bacteria (LAB) in all fermented dough samples were significantly affected by fermentation time (*p* < 0.05). In all dough recipes, LAB counts increased steadily until 120 h of fermentation, and then started decreasing as fermentation time increased, probably due to limitation of nutrients such as lack of fermentable sugar and amino acids in the fermenting matrix (Nsogning et al. 2018; Rawat 2015). Similar findings were reported by Kitessa et al. (2022) during the fermentation of Shameta, a traditional Ethiopian cereal‐based fermented porridge.

#### 3.1.3 Total Yeast and Mold Counts

. The growth characteristics of yeasts and molds in the various dough recipes is presented in Figure 2a–d. Yeast counts increased steadily until 72 h of fermentation across all dough recipes. The growth was consistent throughout the fermentation period, with higher counts noted in the later stages. The increase in yeast counts at the later stages of fermentation might be due to the favorable acidic environment created in the fermenting matrix by LAB (Preetha and Narayanan 2020). In contrast, mold counts increased until 72 h and then declined to zero at the later stages of fermentation. This decline in mold counts at the later stages of fermentation might be due to the lower pH and the production of antimicrobial compounds by lactic acid bacteria and yeasts (Hamad 2012). Similar findings were reported during the fermentation of other African fermented products such as Shameta (Kitessa et al. 2022), Kenkey (Jespersen et al. 1994) and Ogi (Omemu et al. 2007).

### 3.2 Fermentation Kinetics

#### 3.2.1 Changes in pH and Total Titratable Acidity

**FIGURE 3:**
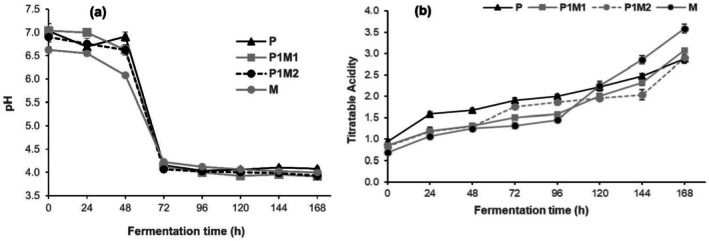
Changes in pH (a) and titrable acidity (b) of pearl millet‐based sourdough recipes. P (100% pearl millet dough); P1M1 (1 pearl millet:1 maize dough); P1M2 (1 pearl millet:2 maize dough), M (100% maize dough). Error bars represent mean ± SD.

Initially, all dough recipes had pH values around 6.6 to 7.0. As fermentation progressed, all samples showed significant decreases in pH (3.91–4.08) at 168 h (Figure 3a). However, there was no significant difference in the pH values of the dough recipes at this fermentation time. In general, the decrease in pH in the various dough samples might result from the growth and metabolic activity of LAB (production of organic acids) during the fermentation process (Ogunsakin et al. 2017). The changes in organic acids such as lactic acid concentrations during fermentation contribute to the distinctive sour flavor of *Injera*, which is influenced by the fermenting medium pH that regulates the production of lactic acid (Jebessa et al. 2024). Additionally, it has been shown that during cereal fermentation, the medium pH is reduced to the level that inhibits the growth of pathogenic bacteria, and also some metabolites which has antimicrobial effects synthesized by LAB during the process (Ojokoh et al. 2015).

The changes in total titratable acidity (TTA) of the dough samples are illustrated in Figure 3b. The TTA of all dough recipes exhibited significant increments with fermentation time. The observed increase in TTA of the sourdough samples might result from the bacterial production of lactic acid and the degradation of carbohydrates into simple sugars and subsequent acidification. Previous studies have also documented a gradual increase in the overall titratable acidity during different cereal‐based fermentations such as maize‐based fermented food products (Mwizerwa et al. 2018), sorghum‐based gluten‐free bread (Almaiman et al. 2021), teff *Injera* dough (Tadesse Bonger et al. 2023), and composite flour dough used for *Injera* preparation (Anberbir et al. 2023).

#### 3.2.2 Changes in Antinutrients

##### 3.2.2.1 Kinetics of Phytate Degradation

**FIGURE 4:**
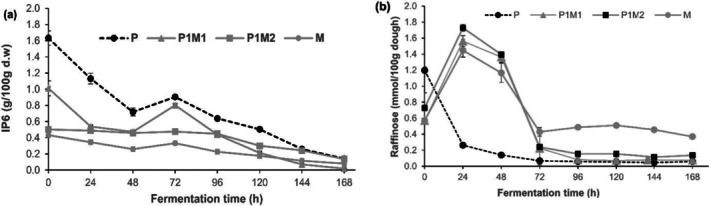
Changes in phytate (a) and raffinose (b) during fermentation of pearl millet‐based sourdough recipes. Error bars represent mean ± SD.

By forming complexes with minerals, proteins, and carbohydrates, phytate reduces their bioavailability and absorption (Feizollahi et al. 2021). In this study, a similar pattern of degradation of phytate (IP6) was observed in all dough fermentations (Figure 4a). There were differences in the phytate content of the various dough recipes at the beginning of fermentation. Pearl millet dough had the highest phytate content (1.63 g/100 g) followed by P1M1 dough (1.01 g/100 g) and P1M2 dough (0.50 g/100 g). As the amount of maize increased in the recipes, the phytate content correspondingly decreased, indicating that much of the phytate content in the recipes is contributed by pearl millet. In general, although the addition of new flour to the fermenting dough samples on the third day of fermentation increased the phytate levels of the dough samples, phytate content decreased by 91.3% in pearl millet dough, by 98.2% in P1M1 dough, and by 72.7% in P1M2 dough after 168 h fermentation. Similar trends in the reduction of phytate content were observed in previous studies of pearl millet fermentation (Eltayeb et al. 2016; Osman 2011). Lactic acid fermentation is known to degrade phytate in cereals through the production of the enzyme phytase by lactic acid bacteria (Endalew et al. 2024; Baye et al. 2013; Osman 2011). Many other factors also affect the extent and rate of phytate degradation in fermented foods, including the endogenous phytase enzyme activities present in raw materials and processing conditions, notably pH, which modulates phytase activities from plants and microbes (Baye et al. 2013; Osman 2011).

##### 3.2.2.2 Change in α‐ Galactooligosaccharides

The α‐ galactooligosaccharides, such as raffinose, are known to cause gastric discomfort primarily due to their resistance to digestion in the human gastrointestinal tract and fermentation in the colon, as well as hindering the digestibility of proteins and bioavailability of minerals (Adebo et al. 2022). Figure 4b shows the changes in concentrations of raffinose (mmol/100 g dough) during fermentation of the various dough types throughout the 168 h fermentation time. Initially, all dough recipes exhibited low raffinose levels, with an increase between 24 and 48 h, particularly in P1M1 and P1M2, following which raffinose levels decreased across all dough recipes, especially after 72 h, and then stabilized at low values by 144–168 h. The highest raffinose degradation was noticed in pearl millet dough (95%) followed by P1M1 dough (87.7%) and P1M2 (80.8%) dough. The observed degradation of raffinose during fermentation of the various pearl millet‐based dough recipes agrees with previous reports of reduction in α‐galactosides during cereal fermentation (Baye et al. 2013; Tou et al. 2007). The degradation of α‐ galactooligosaccharides such as raffinose during fermentation is linked to the production of the enzyme α‐galactosidase by lactic acid bacteria (LeBlanc et al. 2004). The highest degradation of raffinose in pearl millet dough, in comparison to the other recipes, might be attributed to the elevated production of the enzyme induced by the higher concentration of raffinose recorded at the beginning of fermentation in this dough relative to the other dough samples (Songré‐Ouattara et al. 2008).

#### 3.2.3 Changes in Monosaccharide and Disaccharide Levels

**FIGURE 5:**
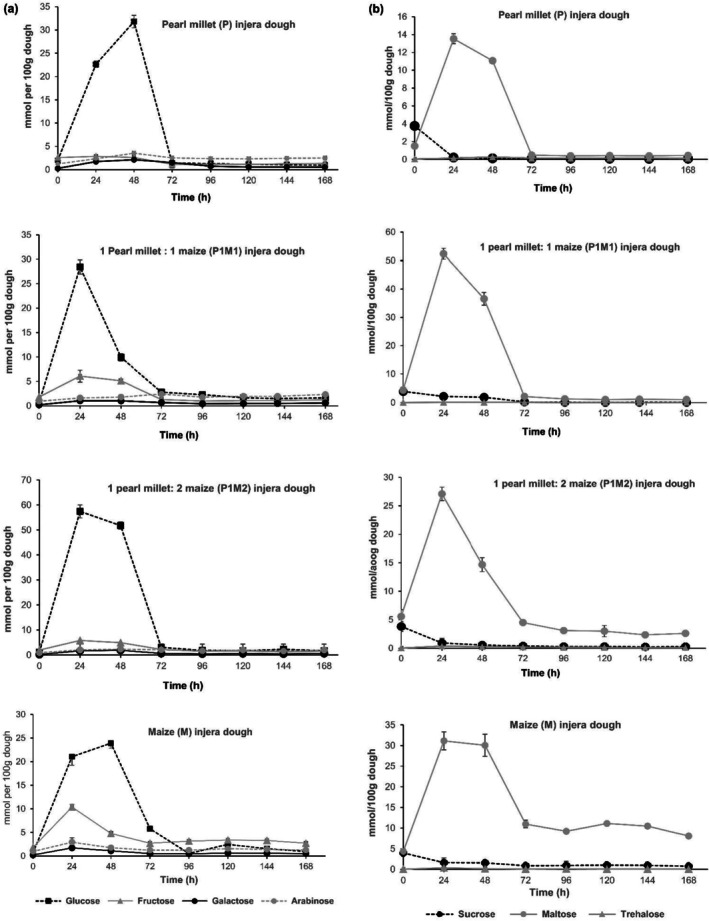
Changes in mono‐ (a), di‐saccharides (b) levels during fermentation of pearl millet‐based dough recipes. Error bars represent mean ± SD.

At the beginning, maltose was the dominant sugar in P1M1, P1M2, and Maize dough samples with values of 4.33, 5.57, and 4.33 mM/100 g respectively (Figure 5a,b). As fermentation progressed, glucose became the main fermentable sugar in P and P1M2 dough samples followed by maltose. On the other hand, maltose continued to be the main fermentable sugar in P1M1 dough followed by glucose. Much of the maltose content at the initial stage of fermentation in P1M1 dough seems to be contributed by maize flour which corresponds to the preparation of this dough by the local community. Maize flour is fermented for the first 2 days and an equal proportion of pearl millet flour is added to the fermented maize dough on the third fermentation day. In general, a rapid increase in maltose concentration was recorded for all dough samples during the first 24 h fermentation after which it decreased to reach a final value of 0.41, 1.06, 2.35, and 8.08 mM/100 g for P, P1M1, P1M2, and M dough samples respectively. Similarly, during the first 48 h of fermentation, the glucose contents of the dough samples increased from an initial range of 0.63–2.01 mM/100 g to a peak of 28.4–57.4 mM/100 g. Subsequently, the glucose content decreased to a final range of 0.94–1.76 mM/100 g.

The increase in soluble sugars during the early fermentation period of the dough samples could be due to the digestion of starch by α‐ and β‐amylases present in the flours and produced by the microbes, while the decreases in these sugars during the later stage of fermentation may result from the utilization of the sugars by microbes (Almaiman et al. 2021; Baye et al. 2013; Osman 2011; Tou et al. 2007). The concentrations of sucrose, fructose, galactose, arabinose, and trehalose were relatively low in all dough recipes and decreased to only trace amounts during the first 72 h of fermentation.

#### 3.2.4 Changes in Organic Acids and Alcohol

**FIGURE 6:**
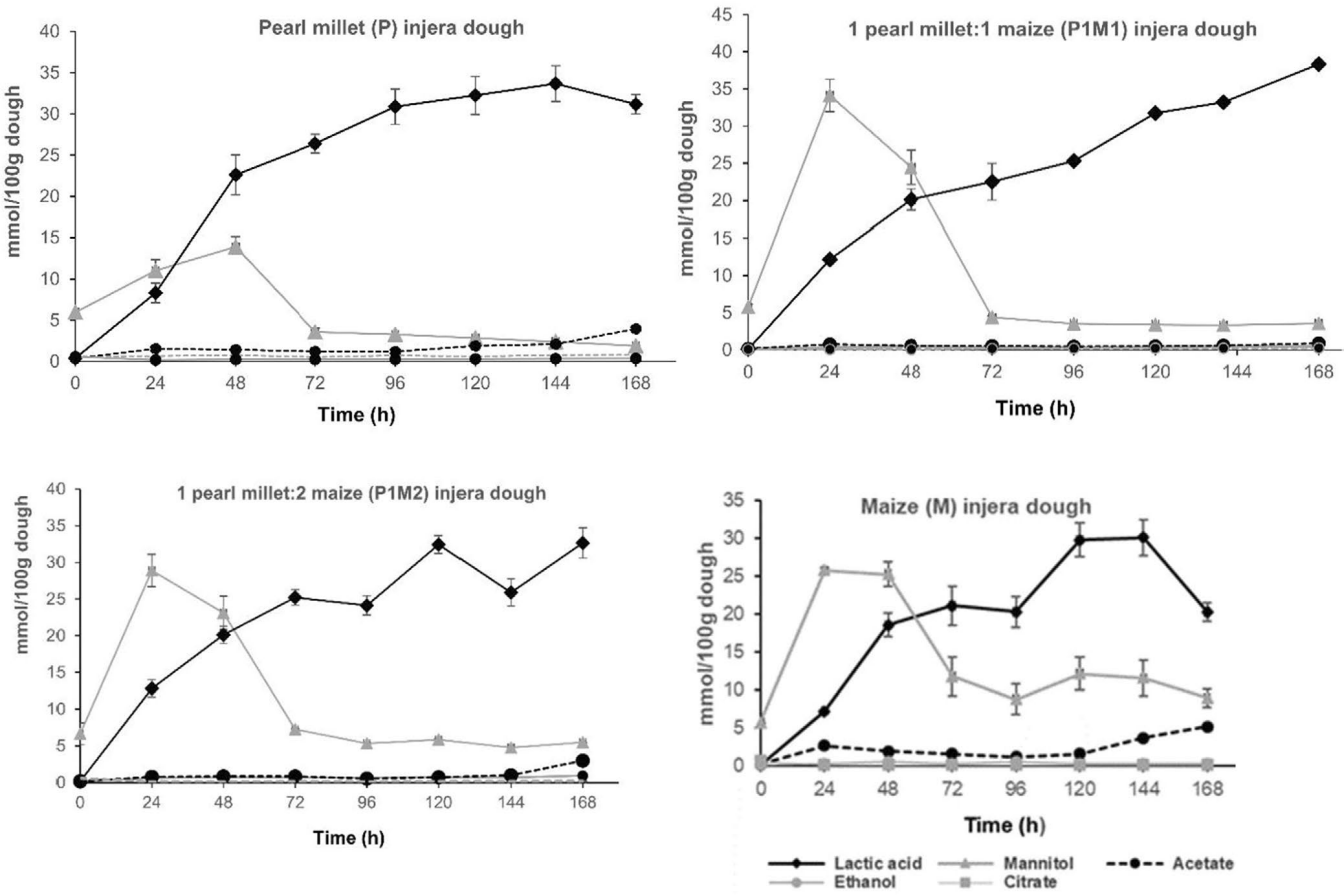
Ethanol, mannitol, lactic acid, acetate, and citrate during fermentation of pearl millet‐based dough recipes. Error bars represent mean ± SD.

Figure 6 depicts changes in the levels of organic acids and alcohol during pearl millet and pearl millet‐maize composite dough fermentations. The fermentation of all the dough recipes can be characterized as a two‐step fermentation, which agrees with other studies (Baye et al. 2013; Lee et al. 2021). In the initial phase, occurring within the first 48 h, heterolactic lactic acid bacteria (LAB) actively metabolized available carbohydrates, leading to the production of lactic acid, mannitol, and small quantities of ethanol. This phase was marked by a rapid increase in lactic acid concentrations alongside transient mannitol accumulation, indicating that LAB utilized sugars, including fructose, as fermentation substrates (Baye et al. 2013; Lee et al. 2021). Following this initial phase, fermentation dynamics shifted towards predominately homolactic fermentation, where LAB primarily converted glucose into lactic acid. This transition was characterized by a steady rise in lactic acid levels, while mannitol concentrations declined, reflecting a change in microbial metabolism and substrate utilization (Tou et al. 2007; Petrova and Petrov 2020). Such findings underscore the complex interactions among microbial populations and their metabolic pathways during the fermentation of cereals, highlighting the significant role of fermentation conditions and substrate composition in influencing the fermentation process (Bhatt et al. 2013). Ethanol, acetate, and citrate were present in very low concentrations. In general, the increased production of lactic acid during dough fermentation is expected to generate antimicrobial compounds that inhibit pathogenic microbes, improve food digestibility by reducing high‐chain carbohydrates, and enhance the bioavailability of essential micronutrients such as iron, zinc, and calcium (Petrova and Petrov 2020).

## 4 Conclusion

Pearl millet and pearl millet‐maize composite dough recipes showed distinct fermentation patterns, significantly impacting their final composition. As fermentation time increased, a notable decrease in pH and substantial increases in total titratable acidity (TTA) were observed across the dough samples. These changes correlated with significant reductions in phytate and raffinose levels in all dough recipes. However, further study is required to assess the bioaccessibility and/or bioavailability of minerals and other nutrients in *Injera* prepared from these dough recipes.

## Author Contributions

**Tadesse Fenta Yehuala:** conceptualization (equal), formal analysis (equal), investigation (equal), methodology (equal), writing – original draft (equal). **Minaleshewa Atlabachew:** conceptualization (equal), formal analysis (equal), methodology (equal), resources (equal), supervision (equal), writing – review and editing (equal). **Mohamad Farshard Aslam:** formal analysis (equal), writing – review and editing (equal). **Lara Allen:** resources (equal), supervision (equal), writing – review and editing (equal). **Howard Griffith:** resources (equal), supervision (equal), writing – review and editing (equal). **Jane L. Ward:** formal analysis (equal), writing – review and editing (equal). **Peter R. Shewry:** formal analysis (equal), writing – review and editing (equal). **Anastasia Kanellou:** supervision (equal), writing – review and editing (equal). **Wanjiku Gichohi‐Wainaina:** supervision (equal), writing – review and editing (equal). **Helen Walle Endalew:** supervision (equal), writing – review and editing (equal). **Metadel Kassahun Abera:** supervision (equal), writing – review and editing (equal). **Mesfin Wogayehu Tenagashaw:** supervision (equal), writing – review and editing (equal). **Gizaw Desta Gessesse:** supervision (equal), writing – review and editing (equal). **Hirut Assaye Cherie:** conceptualization (equal), formal analysis (equal), methodology (equal), resources (equal), supervision (equal), writing – review and editing (equal).

## Ethics Statement

The authors have nothing to report.

## Conflicts of Interest

The authors declare no conflicts of interest.

## Supporting information


Data S1.

## Data Availability

Data will be made available on request from the corresponding author.

## References

[fsn370598-bib-0001] Abdu, A. O. , D. B. Kumssa , E. J. Joy , et al. 2022. “Estimates of Dietary Mineral Micronutrient Supply From Staple Cereals in Ethiopia at a District Level.” Nutrients 14, no. 17: 3469. 36079728 10.3390/nu14173469 PMC9459787

[fsn370598-bib-0002] Adebo, J. A. , P. B. Njobeh , S. Gbashi , et al. 2022. “Fermentation of Cereals and Legumes: Impact on Nutritional Constituents and Nutrient Bioavailability.” Fermentation 8, no. 2: 2.

[fsn370598-bib-0003] Adeyemo, S. M. , and A. A. Onilude . 2013. “Enzymatic Reduction of Anti‐Nutritional Factors in Fermenting Soybeans by *Lactobacillus plantarum* Isolates From Fermenting Cereals.” Nigerian Food Journal 31, no. 2: 84–90.

[fsn370598-bib-0004] Ali, M. A. , A. H. El Tinay , and A. H. Abdalla . 2003. “Effect of Fermentation on the in Vitro Protein Digestibility of Pearl Millet.” Food Chemistry 80, no. 1: 51–54. 10.1016/S0308-8146(02)00234-0.

[fsn370598-bib-0005] Almaiman, S. A. , I. Abdel Rahman , M. Gassem , et al. 2021. “Biochemical Changes During Traditional Fermentation of Saudi Sorghum (*Sorghum bicolor* L.) Cultivars Flour Into Khamir (Local Gluten Free Bread).” Journal of Oleo Science 70, no. 3: 409–415. 10.5650/jos.ess20311. 33658469

[fsn370598-bib-0006] Anberbir, S. M. , N. Satheesh , A. A. Abera , et al. 2023. “Effect of Blending Ratio and Fermentation Time on the Physicochemical, Microbiological, and Sensory Qualities of Injera From Teff, Pearl Millet, and Buckwheat Flours.” CyTA—Journal of Food 21, no. 1: 217–236. 10.1080/19476337.2023.2188058.

[fsn370598-bib-0007] AOAC . 1995. Official Methods of Analysis of AOAC International. AOAC.

[fsn370598-bib-0008] Ashagrie, Z. 2012. “Improvement of Injera Shelf Life Through the Use of Chemical Preservatives.” African Journal of Food, Agriculture, Nutrition and Development 12, no. 5: 6409–6423.

[fsn370598-bib-0009] Baye, K. , C. Mouquet‐Rivier , C. Icard‐Vernière , I. Rochette , and J.‐P. Guyot . 2013. “Influence of Flour Blend Composition on Fermentation Kinetics and Phytate Hydrolysis of Sourdough Used to Make Injera.” Food Chemistry 138, no. 1: 430–436. 10.1016/j.foodchem.2012.10.075. 23265508

[fsn370598-bib-0010] Belay, A. , D. Gashu , E. J. Joy , et al. 2022. “Mineral Micronutrient Status and Spatial Distribution Among the Ethiopian Population.” British Journal of Nutrition 128, no. 11: 2170–2180. 35109956 10.1017/S0007114522000319 PMC9661372

[fsn370598-bib-0011] Berhanu, T. , W. Beshir , and A. Lakew . 2020. “Effect of Integrated Technologies on Production and Productivity of Pearl Millet in the Dryland Areas of Wag Himira Administrative Zone, Eastern Amhara, Ethiopia.” International Journal of Agronomy 2020, no. 1: 4381870.

[fsn370598-bib-0012] Bhatt, S. M. , A. Mohan , and S. K. Srivastava . 2013. “Challenges in Enzymatic Route of Mannitol Production.” International Scholarly Research Notices 2013, no. 1: 914187. 10.5402/2013/914187. PMC4403613 25969783

[fsn370598-bib-0013] da Silva, N. , M. H. Taniwaki , V. C. A. Junqueira , N. Silveira , M. M. Okazaki , and R. A. R. Gomes . 2018. Microbiological Examination Methods of Food and Water: A Laboratory Manual. CRC Press. 10.1201/9781315165011.

[fsn370598-bib-0014] Eltayeb, M. M. , A. B. Hassn , E. E. Babiker , and M. A. Sulieman . 2016. “Effect of Processing Followed by Fermentation on Antinutritional Factors Content of Pearl Millet (*Pennisetum glaucum* L.) Cultivars.” Pakistan Journal of Nutrition 2: 463–467.

[fsn370598-bib-0015] Endalew, H. W. , M. Atlabachew , S. Karavoltsos , et al. 2024. “Effect of Fermentation on Nutrient Composition, Antinutrients, and Mineral Bioaccessibility of Finger Millet Based Injera: A Traditional Ethiopian Food.” Food Research International 190: 114635. 10.1016/j.foodres.2024.114635. 38945624

[fsn370598-bib-0016] Feizollahi, E. , R. S. Mirmahdi , A. Zoghi , R. T. Zijlstra , M. S. Roopesh , and T. Vasanthan . 2021. “Review of the Beneficial and Anti‐Nutritional Qualities of Phytic Acid, and Procedures for Removing It From Food Products.” Food Research International 143: 110284. 10.1016/J.FOODRES.2021.110284. 33992384

[fsn370598-bib-0017] Gowda, N. N. , K. Siliveru , P. V. Prasad , Y. Bhatt , B. P. Netravati , and C. Gurikar . 2022. “Modern Processing of Indian Millets: A Perspective on Changes in Nutritional Properties.” Food 11, no. 4: 499. 10.3390/foods11040499 PMC8871339 35205975

[fsn370598-bib-0018] Hamad, S. H. 2012. “Factors Affecting the Growth of Microorganisms in Food.” Progress in Food Preservation 4: 427. 10.1002/9781119962045.ch20.

[fsn370598-bib-0019] Jebessa, T. F. , G. N. Tolesa , G. Bultosa , S. Abera , and G. G. Hailu . 2024. “A Comprehensive Study on the Effect of Fermentation Time, Baking Temperature, and Baking Time on the Physicochemical and Nutritional Properties of Injera Teff (Eragrostis Teff).” Food and Humanity 2: 100256. 10.1016/j.foohum.2024.100256.

[fsn370598-bib-0020] Jespersen, I. , M. Halm , K. Kpodo , and M. Jakobsen . 1994. “Significance of Yeasts and Moulds Occurring in Maize Dough Fermentation for Kenkey Production.” International Journal of Food Microbiology 24: 239–248. 7703017 10.1016/0168-1605(94)90122-8

[fsn370598-bib-0021] Kitessa, D. A. , K. Bacha , Y. B. Tola , M. Murimi , S. Gershe , and M. Guta . 2022. “Microbial Quality and Growth Dynamics in Shameta: A Traditional Ethiopian Cereal‐Based Fermented Porridge.” Fermentation 8: 1–18.

[fsn370598-bib-0022] LeBlanc, J. G. , M. S. Garro , and G. Savoy de Giori . 2004. “Effect of pH on *Lactobacillus fermentum* Growth, Raffinose Removal, α‐Galactosidase Activity and Fermentation Products.” Applied Microbiology and Biotechnology 65: 119–123. 14727095 10.1007/s00253-003-1532-z

[fsn370598-bib-0023] Lee, S.‐J. , H.‐S. Jeon , J.‐Y. Yoo , and J.‐H. Kim . 2021. “Some Important Metabolites Produced by Lactic Acid Bacteria Originated From Kimchi.” Food 10, no. 9: 2148. 10.3390/foods10092148. PMC8465840 34574257

[fsn370598-bib-0024] McKie, V. A. , and B. V. McCleary . 2016. “A Novel and Rapid Colorimetric Method for Measuring Total Phosphorus and Phytic Acid in Foods and Animal Feeds.” Journal of AOAC International 99, no. 3: 738–743. 10.5740/jaoacint.16-0029. 27076114

[fsn370598-bib-0025] Mihrete, Y. , and G. Bultosa . 2017. “The Effect of Blending Ratio of Tef [*Eragrostis tef* (Zucc) Trotter], Sorghum (*Sorghum bicolor* (L.) Moench) and Faba Bean (*Vicia faba*) and Fermentation Time on Chemical Composition of Injera.” Journal of Nutrition & Food Sciences 7, no. 2: 1–7.

[fsn370598-bib-0026] Mwizerwa, H. , G. O. Abong , S. K. Mbugua , M. W. Okoth , P. Gacheru , and M. Muiru . 2018. “Profiling of Microbial Content and Growth in Fermented Maize‐Based Products From Western Kenya.” Current Research in Nutrition and Food Science Journal 6, no. 2: 509–519.

[fsn370598-bib-0027] Neela, S. , and S. W. Fanta . 2020. “Injera (An Ethnic, Traditional Staple Food of Ethiopia): A Review on Traditional Practice to Scientific Developments.” Journal of Ethnic Foods 7, no. 1: 32. 10.1186/s42779-020-00069-x.

[fsn370598-bib-0028] Niu, Y. , Y. Guo , R. Huang , et al. 2025. “Fermentative Profile and Bacterial Community Structure of Whole‐Plant Triticale Silage (Triticosecale Wittmack) With or Without the Addition of Streptococcus Bovis and Lactiplantibacillus Plantarum.” mSphere 10: e00894. 39873502 10.1128/msphere.00894-24 PMC11852913

[fsn370598-bib-0029] Nsogning, S. D. , S. Fischer , and T. Becker . 2018. “Investigating the Fermentation Behavior of Six Lactic Acid Bacteria Strains in Barley Malt Wort Reveals Limitations in Key Amino Acids and Buffer Capacity.” Food Microbiology 73: 245–253. 10.1016/j.fm.2018.01.010. 29526209

[fsn370598-bib-0030] Ogunsakin, A. O. , V. Vanajakshi , K. A. Anu‐Appaiah , et al. 2017. “Evaluation of Functionally Important Lactic Acid Bacteria and Yeasts From Nigerian Sorghum as Starter Cultures for Gluten‐Free Sourdough Preparation.” LWT—Food Science and Technology 82: 326–334. 10.1016/j.lwt.2017.04.048.

[fsn370598-bib-0031] Ojokoh, A. O. , O. E. Fayemi , F. C. K. Ocloo , and F. I. Nwokolo . 2015. “Effect of Fermentation on Proximate Composition, Physicochemical and Microbial Characteristics of Pearl Millet (*Pennisetum glaucum* (L.) R. Br.) and Acha (*Digitaria exilis* (Kippist) Stapf) Flour Blends.” Journal of Agricultural Biotechnology and Sustainable Development 7, no. 1: 1–8. 10.5897/JABSD2014.0236.

[fsn370598-bib-0032] Omemu, A. M. , O. B. Oyewole , and M. O. Bankole . 2007. “Significance of Yeasts in the Fermentation of Maize for Ogi Production.” Food Microbiology 24: 571–576. 17418307 10.1016/j.fm.2007.01.006

[fsn370598-bib-0033] Osman, M. A. 2011. “Effect of Traditional Fermentation Process on the Nutrient and Antinutrient Contents of Pearl Millet During Preparation of Lohoh.” Journal of the Saudi Society of Agricultural Sciences 10, no. 1: 1–6. 10.1016/J.JSSAS.2010.06.001.

[fsn370598-bib-0034] Petrova, P. , and K. Petrov . 2020. “Lactic Acid Fermentation of Cereals and Pseudocereals: Ancient Nutritional Biotechnologies With Modern Applications.” Nutrients 12, no. 4: 1118. 10.3390/nu12041118. 32316499 PMC7230154

[fsn370598-bib-0035] Petrović, T. Ž. , V. M. Tomović , S. Kocić‐Tanackov , et al. 2025. “Microbial Dynamics and Quality Evolution in the Spontaneous Fermentation of the Traditional Meat Product Sjenica Sheep Stelja.” Fermentation 11, no. 4: 221.

[fsn370598-bib-0036] Preetha, S. S. , and R. Narayanan . 2020. “Factors Influencing the Development of Microbes in Food.” Shanlax International Journal of Arts, Science and Humanities 7, no. 3: 57–77. 10.34293/sijash.v7i3.473.

[fsn370598-bib-0037] Rani, S. , R. Singh , R. Sehrawat , B. P. Kaur , and A. Upadhyay . 2018. “Pearl Millet Processing: A Review.” Nutrition & Food Science 48, no. 1: 30–44. 10.1108/NFS-04-2017-0070.

[fsn370598-bib-0038] Ravyts, F. , and L. De Vuyst . 2011. “Prevalence and Impact of Single‐Strain Starter Cultures of Lactic Acid Bacteria on Metabolite Formation in Sourdough.” Food Microbiology 28, no. 6: 1129–1139. 10.1016/j.fm.2011.03.004. 21645811

[fsn370598-bib-0039] Rawat, S. 2015. “Food Spoilage: Microorganisms and Their Prevention.” Asian Journal of Plant Science and Research 5: 47–56.

[fsn370598-bib-0046] Sadler, G. D. , and P. A. Murphy . 2010. “pH and Titratable Acidity.” Food Analysis 4: 219–238.

[fsn370598-bib-0040] Satyavathi, C. T. , S. Ambawat , V. Khandelwal , and R. K. Srivastava . 2021. “Pearl Millet: A Climate‐Resilient Nutricereal for Mitigating Hidden Hunger and Provide Nutritional Security.” Frontiers in Plant Science 12: 659938. 10.3389/fpls.2021.659938. 34589092 PMC8475763

[fsn370598-bib-0041] Shewry, P. R. , A. H. P. America , A. Lovegrove , et al. 2022. “Comparative Compositions of Metabolites and Dietary Fiber Components in Doughs and Breads Produced From Bread Wheat, Emmer, and Spelt and Using Yeast and Sourdough Processes.” Food Chemistry 374: 131710. 10.1016/j.foodchem.2021.131710. 34891089 PMC8759010

[fsn370598-bib-0042] Songré‐Ouattara, L. T. , C. Mouquet‐Rivier , C. Icard‐Vernière , C. Humblot , B. Diawara , and J. P. Guyot . 2008. “Enzyme Activities of Lactic Acid Bacteria From a Pearl Millet Fermented Gruel (Ben‐Saalga) of Functional Interest in Nutrition.” International Journal of Food Microbiology 128, no. 2: 395–400. 18937991 10.1016/j.ijfoodmicro.2008.09.004

[fsn370598-bib-0043] Tadesse Bonger, Z. , M. Kassahun Abera , T. A. Habitu , et al. 2023. “Isolation and Identification of Dominant Lactic Acid Bacteria and Yeast Species From Teff (Eragrostis Teff) Injera Dough Fermentation.” CyTA—Journal of Food 21, no. 1: 718–734. 10.1080/19476337.2023.2276830.

[fsn370598-bib-0044] Tou, E. H. , C. Mouquet‐Rivier , C. Picq , A. S. Traorè , S. Trèche , and J. P. Guyot . 2007. “Improving the Nutritional Quality of Ben‐Saalga, a Traditional Fermented Millet‐Based Gruel, by Co‐Fermenting Millet With Groundnut and Modifying the Processing Method.” LWT—Food Science and Technology 40, no. 9: 1561–1569. 10.1016/j.lwt.2006.12.001.

[fsn370598-bib-0045] Yadav, D. N. , M. Sharma , N. Chikara , T. Anand , and S. Bansal . 2014. “Quality Characteristics of Vegetable‐Blended Wheat–Pearl Millet Composite Pasta.” Agricultural Research 3: 263–270.

