# Supplementary material for: Fermentation Kinetics and Changes in Levels of Antinutrients in Pearl Millet and Pearl Millet‐Maize Composite Dough Recipes Used to Prepare Injera
Source: Food Sci Nutr. 2025 Jul 9;13(7):e70598. doi: 10.1002/fsn3.70598 (PMC12241443; doi:10.1002/fsn3.70598)
Supplement: Supplementary file 1 — Data S1. [file FSN3-13-e70598-s001.doc]

**Supplementary materials**

SM Table 1

Changes in microbial counts during pearl millet-based dough fermentations.

| Fermentation time (h) | TAP | LAB | Yeast | Mold |
| --- | --- | --- | --- | --- |
|  | Pearl millet (P) dough | |  |  |
| 0 | 3.90±0.90^a^ | 2.06±0.84^a^ | 1.30±0.06^a^ | 1.04±0.38^b^ |
| 24 | 8.56±0.96^bc^ | 5.39±0.87^b^ | 3.15±0.51^a^ | 3.30±0.23^b^ |
| 48 | 9.11±0.93^c^ | 8.92±0.80^de^ | 5.25±0.33^b^ | 3.96±0.21^bc^ |
| 72 | 8.80±1.13^c^ | 9.12±0.91^e^ | 7.43±0.30^e^ | 5.48±0.34^d^ |
| 96 | 8.78±1.05^c^ | 8.97±0.81^b^ | 6.60±0.45^cd^ | 4.94±0.27^cd^ |
| 120 | 8.00±1.03^ab^ | 8.95±0.11^de^ | 6.54±0.44^cd^ | 4.66±0.46^b^ |
| 144 | 7.86±0.91^ab^ | 8.64±0.82^c^ | 7.01±0.53^de^ | 3.98±0.23^cd^ |
| 168 | 5.16±0.5^a^ | 5.79±0.89^cd^ | 6.00±0.31^c^ | ND |
|  | 1 Pearl millet: 1 Maize (P1M1) dough | | |  |
| 0 | 3.57±0.90^bc^ | 2.09±0.82^a^ | 1.81±0.51^a^ | 1.77±0.30^bc^ |
| 24 | 8.94±0.91^c^ | 5.72±0.80^c^ | 5.67±0.89^b^ | 4.00±0.32^bc^ |
| 48 | 8.90±0.90^c^ | 8.63±0.92^c^ | 7.54±0.33^c^ | 3.97±0.23^bc^ |
| 72 | 8.74±0.93^bc^ | 8.43±0.83^c^ | 8.00±0.32^c^ | 5.15±0.41^d^ |
| 96 | 8.58±0.96^bc^ | 8.52±0.80^c^ | 7.93±0.29^c^ | 5.43±0.38^d^ |
| 120 | 7.49±1.14^abc^ | 8.60±0.92^c^ | 7.79±0.39^c^ | 3.87±0.32^bc^ |
| 144 | 7.29±1.03^ab^ | 8.32±0.85^c^ | 8.17±0.35^c^ | 3.24±0.29^bc^ |
| 168 | 5.04±1.04^a^ | 4.78±1.05^b^ | 7.60±0.41^c^ | ND |
|  | 1 Pearl millet: 2 Maize (P1M2) dough | | |  |
| 0 | 3.54±1.01^ab^c | 2.40±0.67^a^ | 1.56±0.46^a^ | 1.07±0.24^ab^ |
| 24 | 8.90±1.10^c^ | 5.66±0.81^bc^ | 5.15±0.39^b^ | 2.98±0.23^ab^ |
| 48 | 9.10±0.93^c^ | 8.59±0.91^bc^ | 7.02±0.55^c^ | 5.50±0.71^b^ |
| 72 | 8.75±0.98^c^ | 8.46±0.89^bc^ | 8.01±0.33^de^ | 4.97±0.23^b^ |
| 96 | 8.50±0.93^bc^ | 8.86±0.76^c^ | 7.94±0.28^de^ | 4.95±0.22^b^ |
| 120 | 7.52±0.91^ab^ | 8.27±0.85^b^ | 7.84±0.41^d^ | 3.51±0.94^ab^ |
| 144 | 7.48±1.03^ab^ | 8.23±0.74^b^ | 8.59±0.45^e^ | 4.35±0.33^b^ |
| 168 | 4.40±0.88^a^ | 5.20±0.91^b^ | 8.38±0.73^de^ | ND |
|  | Maize (M) dough | |  |  |
| 0 | 3.16±0.82^ab^ | 2.44±0.73^a^ | 1.76±0.46^a^ | 1.00±0.32^ab^ |
| 24 | 8.92±1.11^b^ | 5.62±0.97^bc^ | 5.14±0.39^b^ | 3.07±0.24^ab^ |
| 48 | 9.10±0.96^b^ | 8.54±1.31^bc^ | 6.49±0.42^c^ | 5.42±0.56^b^ |
| 72 | 8.76±1.12^b^ | 8.51±0.95^bc^ | 7.99±0.31^d^ | 4.96±0.25^b^ |
| 96 | 8.40±0.84^ab^ | 8.92±0.86^c^ | 7.92±0.36^d^ | 4.98±0.85^b^ |
| 120 | 7.50±1.18^a^ | 8.04±0.86^b^ | 7.88±0.37^d^ | 3.56±0.52^b^ |
| 144 | 7.52±1.21^a^ | 7.10±0.95^b^ | 8.63±0.37^d^ | 2.89±0.37^b^ |
| 168 | 5.42±1.08^a^ | 5.99±0.03^b^ | 8.40±0.33^d^ | ND |

TPC-total plate count; LAB-lactic acid bacterial count; ND-non-detectable; data shown in mean ± standard deviation, (n = 3 replicates). Mean with different letters across columns were significantly different

SM Table 2

pH and total titrable acidity (TTA) level of fermented pearl millet injera sourdough.

|  | **Pearl millet (P)** | | **1 pearl millet: 1maize (P1M1)** | | **1 pearl millet: 2 maize (P1M2)** | | **Maize (M)** | | **pH percent reduction** | | | |
| --- | --- | --- | --- | --- | --- | --- | --- | --- | --- | --- | --- | --- |
| **Fermentation time (h)** | *pH* | *TTA* | *pH* | *TTA* | *pH* | *TTA* | *pH* | *TTA* | P | P1M1 | (P1M2 | M |
| **0** | 7.03 ±0.16^c^ | 0.95±0.05^a^ | 7.04±0.03^d^ | 0.86±0.04^a^ | 6.90±0.11^c^ | 0.83±0.04^a^ | 6.62±0.07^e^ | 0.67±0.02^a^ | 0 | 0 | 0 | 0 |
| **24** | 6.69±0.10^b^ | 1.59±0.02^b^ | 7.00±0.06^d^ | 1.20±0.06^ab^ | 6.75±0.12^bc^ | 1.18±0.10^b^ | 6.55±0.04^d^ | 1.07±0.02^b^ | 4.84 | 0.57 | 2.17 | 1.06 |
| **48** | 6.19±0.10^bc^ | 1.67±0.03^bc^ | 6.62±0.07^c^ | 1.32±0.04^b^ | 6.62±0.11^b^ | 1.31±0.05^b^ | 6.08±0.02^ab^ | 1.25±0.01^bc^ | 12.0 | 5.97 | 4.06 | 8.16 |
| **72** | 4.15±0.01^a^ | 1.90±0.06^cd^ | 4.13±0.03^b^ | 1.51±0.03^b^ | 4.07±0.03^a^ | 1.76±0.04^c^ | 4.22±0.02^c^ | 1.32±0.07^c^ | 41.0 | 41.3 | 41.0 | 36.3 |
| **96** | 4.04±0.03^a^ | 2.01±0.05^de^ | 4.00±0.05^a^ | 1.58±0.04^b^ | 4.02±0.01^a^ | 1.87±0.07^cd^ | 4.12±0.02^b^ | 1.45±0.04^c^ | 43.2 | 43.2 | 41.7 | 37.8 |
| **120** | 4.06±0.01^a^ | 2.22±0.09^e^ | 3.92±0.02^a^ | 2.01±0.03^c^ | 4.00±0.01^a^ | 1.96±0.01^cd^ | 4.06±0.02^ab^ | 2.25±0.10^d^ | 42.3 | 44.3 | 42.0 | 38.7 |
| **144** | 4.10±0.02^a^ | 2.48±0.06^f^ | 3.95±0.01^a^ | 2.33±0.07^c^ | 3.98±0.03^a^ | 2.04±0.12^d^ | 4.03±0.02^a^ | 2.86±0.10^e^ | 41.7 | 43.9 | 42.3 | 39.1 |
| **168** | 4.08±0.03^a^ | 2.88±0.10^g^ | 3.91±0.02^a^ | 3.08±0.06^d^ | 3.93±0.03^a^ | 2.90±0.13^e^ | 4.00±0.02^a^ | 3.59±0.10^f^ | 42.0 | 44.5 | 43.0 | 39.6 |

The values are in mean and standard deviation (with triplicate experiments). Mean with different letters across columns were significantly different (*p* < .05).
